# The study protocol for PREDICT AF RECURRENCE: a PRospEctive cohort stuDy of surveIllanCe for perioperaTive Atrial Fibrillation RECURRENCE in major non-cardiac surgery for malignancy

**DOI:** 10.1186/s12872-018-0862-9

**Published:** 2018-06-26

**Authors:** Satoshi Higuchi, Yusuke Kabeya, Kenichi Matsushita, Keisei Tachibana, Riken Kawachi, Hidefumi Takei, Yutaka Suzuki, Nobutsugu Abe, Yorihisa Imanishi, Kiyoshi Moriyama, Tomoko Yorozu, Koichiro Saito, Masanori Sugiyama, Haruhiko Kondo, Hideaki Yoshino

**Affiliations:** 1 Division of Cardiology, Department of Internal Medicine II, Kyorin University School of Medicine, Tokyo, Japan; 2 Division of General Internal Medicine, Department of Internal Medicine, Tokai University, Isehara, Kanagawa Japan; 3 Department of Home Care Medicine, Saiyu Clinic, Saitama, Japan; 4 Department of General Thoracic Surgery, Kyorin University School of Medicine, Tokyo, Japan; 5 Department of General Thoracic Surgery, Nihon University School of Medicine, Tokyo, Japan; 6 Department of Surgery, Kyorin University School of Medicine, Tokyo, Japan; 7 Department of Otorhinolaryngology, Head and Neck Surgery, Kawasaki Municipal Kawasaki Hospital, Kawasaki, Kanagawa Japan; 8 Department of Anesthesiology, Kyorin University School of Medicine, Tokyo, Japan; 9 Department of Otolaryngology-Head and Neck Surgery, Kyorin University School of Medicine, Tokyo, Japan; 10 Division of Cardiology, Department of Internal Medicine II, Kyorin University School of Medicine, 6-20-2 Shinkawa, Mitaka City, Tokyo, 181-8611 Japan

**Keywords:** Perioperative atrial fibrillation (POAF), Non-cardiac surgery, Oncology, Stroke

## Abstract

**Background:**

A previous retrospective cohort study established the relationship between perioperative atrial fibrillation (POAF) and subsequent mortality and stroke. However, the details regarding the cause of death and etiology of stroke remain unclear.

**Methods:**

The prospective cohort study of surveillance for perioperative atrial fibrillation recurrence in major non-cardiac surgery for malignancy (PREDICT AF RECURRENCE) registry is an ongoing prospective cohort study to elucidate the long-term recurrence rate and the clinical impact of new-onset POAF in the setting of head and neck, non-cardiac thoracic, and abdominal surgery for malignancy. In this study, cardiologists collaborate with a surgical team during the perioperative period, carefully observe the electrocardiogram (ECG) monitor, and treat arrhythmia as required. Furthermore, patients who develop new-onset POAF are followed up using a long-term Holter ECG monitor, SPIDER FLASH-t AFib^®^, to assess POAF recurrence.

**Discussion:**

Even if patients with malignancy survive by overcoming the disease, they may die from any preventable cardiovascular diseases. In particular, those with POAF may develop cardiogenic stroke in the future. Because details of the natural history of patients with POAF remain unclear, investigating the need to continue anticoagulation therapy for such patients is necessary. This study will provide essential information on the recurrence rate of POAF and new insights into the prediction and treatment of POAF.

**Trial registration:**

University Hospital Medical Information Network Clinical Trial Registry (UMIN-CTR): UMIN000016146; Data of Registration: January 7, 2015.

**Electronic supplementary material:**

The online version of this article (10.1186/s12872-018-0862-9) contains supplementary material, which is available to authorized users.

## Background

Atrial fibrillation (AF) is one of the most common types of arrhythmia and a common health-related problem, with increasing incidence and prevalence worldwide [1, 2]. AF is associated with increased mortality and incidence of thromboembolic events, such as stroke [3–5], and is most likely induced by clinical conditions, such as hypertension, diabetes mellitus, and heart failure [6–8]. Furthermore, AF often occurs after both cardiac and non-cardiac surgeries, usually on the second or third postoperative day [9], with an incidence rate of 10–65% and 1–9%, respectively [9–11]. The massive differences in the reported incidence rate could be attributed to the possible overlooking of short-duration AF and the variable impact of each surgical procedure on the incidence. Although perioperative AF (POAF) seems to be a temporary cardiac event that does not affect the subsequent clinical course, POAF is reported to be associated with long-term mortality and stroke, even in the setting of non-cardiac surgery and general clinical situations [10]. Furthermore, some recent studies demonstrated that malignancy was related to a higher prevalence of AF [12–14]. Notably, Conen et al. demonstrated that the multi-variate adjusted hazard ratio of AF after diagnosis of breast cancer was as high as 4.67 (95% CI, 2.85–7.64) in the first 3 months, regardless of treatment assignments [14].

Despite recognizing AF as an independent risk factor for mortality, the evidence to determine AF as a causal factor is insufficient [15]. Hence, determining whether AF is the direct and decisive cause of death in the general population without any cardiac disease remains doubtful. In cardiac surgery, the frequent occurrence of POAF with impaired cardiac function might induce cardiac events, such as heart failure and cardiac death. However, in non-cardiac surgery, wherein patients do not usually present with such impairment whether AF directly correlates with mortality or only a surrogate marker remains undetermined.

The recent advancements in the treatment of malignancies have enhanced the prognosis of such patients, increasing the subsequent survivor population at the risk of cardiovascular events [16]. Patients with AF recurrence after the perioperative period are susceptible to subsequent embolic events; therefore, both long- and short-term POAF management to prevent adverse clinical events should be considered. Although a previous study [10] suggested that anticoagulation therapy could be beneficial for patients with POAF, it did not identify patients who were at high risk for subsequent stroke and who should start anticoagulation therapy after discharge. If most factors causing stroke are related to atherosclerosis, antiplatelet therapy along with appropriate risk management regimen should be provided. Particularly, patients with malignancy sometimes face complicated treatment choices because of elevated thrombotic and bleeding risks [17]. In such cases, a survey detailing the short- and long-term clinical course of those with POAF might be helpful for subsequent daily clinical management.

### Hypothesis

The present study has been conducted to investigate our primary hypothesis that the patients with new-onset POAF will develop AF recurrence in the future.

## Methods/design

### Study design and population

**Fig. 1:**
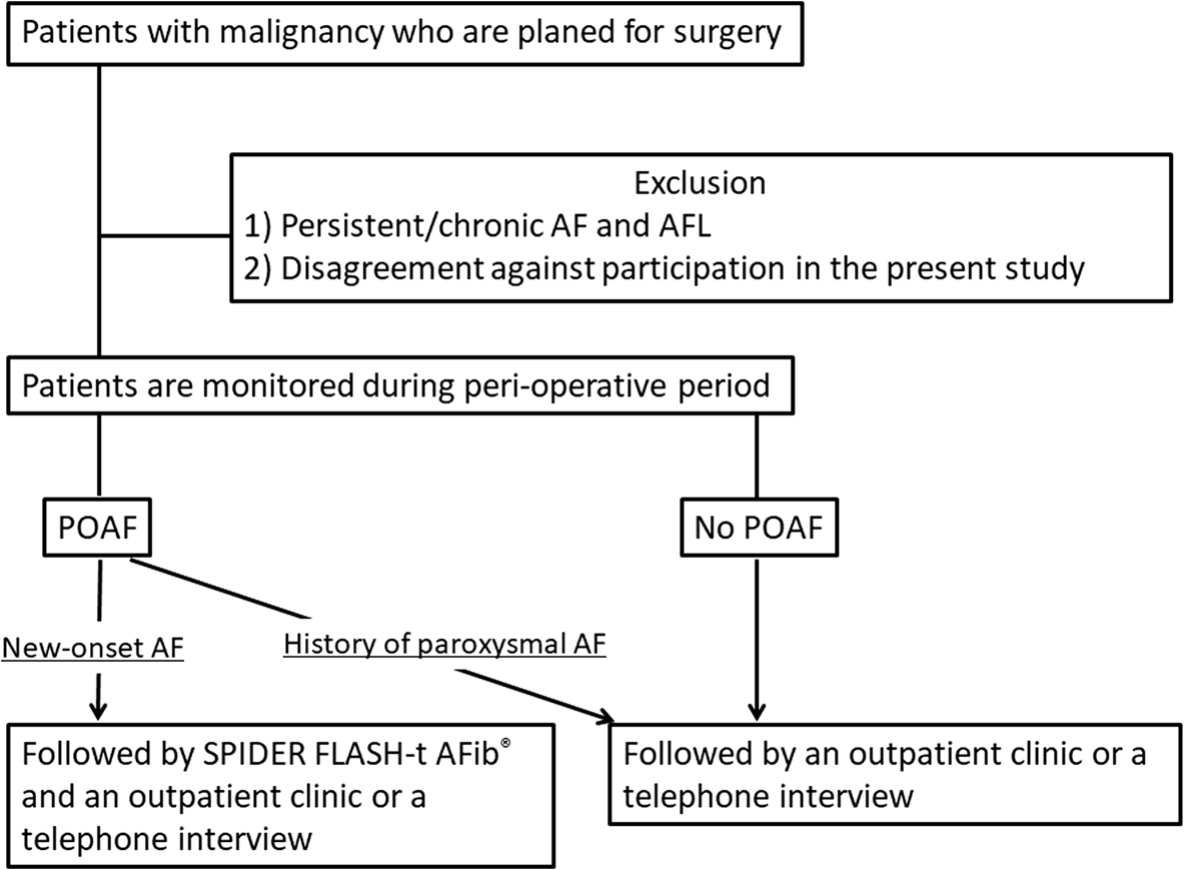
The flowchart of PREDICT AF RECURRENCE. Patients with new-onset AF are followed by using SPIDER FLASH-t AFib®. *AF* Atrial fibrillation, *AFL* Atrial flutter, *POAF* Perioperative atrial fibrillation

The PRospEctive cohort stuDy of surveIllanCe for perioperaTive Atrial Fibrillation RECURRENCE (PREDICT AF RECURRENCE) in major non-cardiac surgery for malignancy is an ongoing prospective, single-center, observational study that is designed to illustrate the clinical impact of POAF on mortality and morbidity as cardiologists collaborate with a surgical team during the perioperative period and investigate the frequency of AF recurrence after discharge in patients with malignancy. In this study, we examined consecutive patients who underwent non-cardiac surgery under general anesthesia due to definitive or suspected malignancy. We followed patients who developed new-onset AF by using a cardiac event recorder. Figure 1 shows the flowchart of the study. The present study enrolled from July 2014 and the registered patients will be followed up until December 2022.

### Inclusion criteria

In this study, we enrolled patients (age: ≥20 and ≤90 years) who planned to undergo surgery in Kyorin University Hospital (Tokyo, Japan) from July 2014. The malignancies registered were as follows: head and neck (e.g., pharyngeal, laryngeal, tongue, mandible, buccal mucosal, gingival, and glottic), chest (e.g., esophageal, lung, and lung metastases), or abdomen (gallbladder, extra- and intrahepatic bile duct, pancreatic, duodenal, Vater papillary, hepatic cell, and liver metastases) cancers. Some previous studies have demonstrated that the prevalence of POAF was frequently associated with the surgery site and, thus, could be infrequent in less-invasive surgical treatments [10, 18]. Hence, we did not include patients with gastric and colon cancers in this study because a majority of them were treated with less-invasive laparoscopic surgery in our institute.

### Exclusion criteria

In this study, we excluded patients with persistent or chronic AF and atrial flutter. Although those with known paroxysmal AF before surgery were included for in-hospital analysis, they were excluded in the analysis of long-term AF recurrence.

### Baseline assessment

The baseline survey was conducted 1–3 days preoperatively by using a study-specific questionnaire for collecting information, including age, sex, current medications, alcohol consumption, smoking status, past histories of thoracotomy and cardiogenic stroke, and complications, such as heart failure, coronary artery disease, peripheral artery diseases, and chronic obstructive pulmonary disease. Table 1 shows the definition of each complication. We performed blood examination within 3 months preoperatively, which included blood cell counts, C-reactive protein (CRP), D-dimer, liver enzyme, electrolytes, serum creatinine, thyroid hormone, and B-type natriuretic peptide (BNP). In addition, we performed a respiratory function test (including forced expiratory volume and forced vital capacity) and 12-lead electrocardiography (ECG) at the same time with blood examination. As a routine clinical practice in the institute, Doppler ultrasonography was performed on patients with D-dimer level of ≥0.5 μg/mL to assess superficial and deep venous thromboses of the lower extremities.

**Table 1:** Definition of complications

Heart Failure	Fulfills any of the following:
	1) Any symptoms such as orthopnea, paroxysmal nocturnal dyspnea, or dyspnea on effort and BNP≥100 pg/mL
	2) A history of admission because of congestive heart failure.
Hypertension	Systolic blood pressure ≥140 mmHg and/or diastolic blood pressure ≥90 mmHg
Diabetes Mellitus	Fulfills 1) and 2)
	1) Fasting plasma glucose ≥126 mg/dL or casual plasma glucose ≥200 mg/dL
	2) HbA1c ≥6.5%
Coronary Artery Disease	Patients with any of the following: LAD, LCx, or RCA with stenosis ≥75%, or LMT with stenosis ≥50% if a patient has undergone coronary angiography or computed tomography.
Peripheral Artery Disease	The term includes carotid artery stenosis/occlusion, intracranial middle- and large-artery stenosis/occlusion, renal stenosis, arteriosclerosis obliterans, and aortic aneurysm, all of which are diagnosed by any imaging modality.
Chronic Obstructive Pulmonary Disease	fulfills 1) and 2)
	1) FEV_1_/FVC ratio <0.7
	2) Predicted to be irreversible after the administration of an inhaled bronchodilator

BNP, B-type natriuretic peptide; LAD, left anterior descending artery; LCx, left circumflex artery; RCA, right coronary artery; LMT, left main trunk; FEV_1_, forced expiratory volume in 1 second; FVC, forced vital capacity

### Data collection during general anesthesia

During the surgery, anesthesiologists provided general care to patients. In addition, heart rate, rhythm, and ST-T changes were continuously monitored by an ECG and appropriately recorded. The final diagnosis of each arrhythmia was confirmed by cardiologists.

### Operation record

We recorded the stage of malignancy, type of surgery, extent of tumor resection and lymph node dissection, and pathological findings in the patients. The stage and extent of lymph node dissection in each surgery were determined based on the Union of International Cancer Control (UICC) TNM classification seventh edition.

### Postoperative data collection

In this study, all patients were observed with an ECG monitor device for a minimum of 24 h up to 30 days postoperatively; the monitoring duration was based on each surgeon’s discretion. In addition, duration of monitoring and surgical intensive care unit stay were recorded. Patients with any type of arrhythmia requiring an intervention were treated by cardiologists in accordance with the current guideline. Furthermore, anticoagulation therapy was provided for those who developed AF if CHA2DS2 VASc score [19] was ≥1 and ≥2 in men and women, respectively, after surgeons assessed the bleeding risk and permitted the prescription. Subsequently, the presence of symptoms, onset time/day, and total duration of AF during hospitalization were recorded, and blood examination, such as white blood cell, hemoglobin, serum creatinine, CRP, and electrolytes, was planned on the following and the fourth postoperative day. We examined the presence of postoperative complications, including myocardial infarction; congestive heart failure; bleeding; thrombosis, such as stroke, transient ischemic attack, ischemic bowel disease, and pulmonary embolism; any infections; acute kidney injury; and desaturation necessary for home oxygen therapy. Finally, echocardiography was immediately performed for patients with new-onset AF.

### Follow-up after discharge

In this ongoing study, the follow-up data would be collected from a face-to-face interview in an outpatient clinic, a telephone interview, or a medical record. The follow-up flow chart is shown in Table 2. Regardless of the presence of POAF, the patients in this study would be followed up for all-cause, cardiac, non-cardiac, and malignancy-related mortality; AF occurrence or recurrence; and stroke. The diagnosis and etiology of stroke in this study were determined by two or more neurologists and radiologists independent from the investigators. The patients who developed new-onset AF were continuously followed up using SPIDER FLASH-t AFib^®^ (LivaNova, London, UK) for approximately 2 weeks after 1 and 12 months postoperatively. The device can record heart rhythm non-invasively for the longest duration in Japan. The dynamic memory in the device allows for rhythm episodes to be accurately identified regardless of their duration. Any arrhythmia, including AF, is recorded automatically (Additional file 1: Table S1). In addition, follow-up was performed after 6 months if required. Follow-up was recorded throughout the day except during the time when patients were taking a bath. Patients who were unable to handle SPIDER FLASH-t AFib^®^ were examined using 24-h Holter ECG for 2 to 4 times. If AF recurrence was documented 1 month postoperatively, the following event monitoring was not planned. The patients who developed a rash because of an electrode seal at the first record could stop the following examination. If the patients were hospitalized at 1, 6, or 12 months postoperatively, they could be evaluated using an ECG monitor device of the residing ward instead of SPIDER FLASH-t AFib^®^. The records acquired by SPIDER FLASH-t AFib^®^ were assessed by an external expert organization, and the final decision was confirmed by two cardiologists. With regard to the 24-h Holter ECG and ECG monitor device in the ward, two cardiologists analyzed and diagnosed the recorded ECG. In the case of differential diagnosis, they attain consensus after sufficient discussion to conclude the final diagnosis. Information on anticoagulation, such as type, dose, duration, and adherence, are collected by cardiologists. Of note, patients without AF recurrence for a year can discontinue anticoagulation treatment.

**Table 2:** Follow-up in chronological order

	1	3	6	12	24	36	48	60	(month)
New-onset POAF									
clinic visit	✔	✔	✔	✔	✔	✔	✔	✔	
blood examination	✔		✔	✔					
event recorder	✔		✔^a^	✔					
12-lead ECG	✔	✔	✔	✔	✔	✔	✔	✔	
No POAF									
clinic visit	✔		✔	✔	✔	✔	✔	✔	
blood examination	✔		✔	✔					
Known PAF prior to surgery									
clinic visit	✔		✔	✔	✔	✔	✔	✔	
blood examination	✔		✔	✔					

^a^: if required

ECG: electrocardiogram; PAF: paroxysmal atrial fibrillation; POAF: perioperative atrial fibrillation

### Outcome definition

Detailed outcomes defined in Additional file 2.

### Short-term outcome

In this study, short-term outcomes comprise new-onset POAF; any arrhythmia; embolic events, such as stroke; and mortality until the thirtieth day after surgery. We defined POAF as AF during and/or after surgery until the thirtieth postoperative day. In addition, we also evaluated the type of arrhythmia, etiology of stroke, and cause of death.

### Long-term outcome

We followed up all patients with and without POAF in this study. Long-term outcomes included AF recurrence (evaluated only in those with new-onset POAF); embolic events, such as stroke; recurrent or de novo malignancy; and mortality on and after the thirty-first postoperative day. New-onset AF occurring on and after the thirty-first day was not considered as POAF, but as “conventional” paroxysmal AF. We defined AF recurrence as repetitive AF lasting ≥30 s on and after the thirty-first day, which was documented by SPIDER FLASH-t AFib^®^, 24-h Holter ECG, or hospital ECG monitor [20]. Notably, patient’s self-assessment and symptoms did not contribute to the definitive diagnosis. Furthermore, the cause of death included cardiac, non-cardiac, and cancer-related mortalities.

### Sub-analysis

We will conduct sub-analyses investigating the predictor of POAF in each malignancy, and the incidence and the characteristics of other arrhythmias.

### Statistical analysis

No preceding study evaluated the incidence of AF recurrence in patients with POAF. Therefore, we assumed the incidence in reference to the first 200 patietns in our study, which indicated a 25% recurrence rate. We assumed a 0.97% incidence of AF in patients without POAF based on a previous study [6]. Based on 80% power and a significance level of 0.05, 38 patients with new-onset POAF were required. Assuming a 15% dropout rate and a 9% incidence rate of POAF, 497 (new-onset POAF, *n* = 45; non-POAF, *n* = 452) were required at least. In this study, the relationship between exposure variables and in-hospital outcomes was assessed using logistic regression analyses and expressed as odds ratio, 95% confidential interval, and *P* value. Variables with *P* <0.10 in the univariate logistic regression analysis were included in the multivariate logistic regression analysis. In addition, we assessed the long-term outcomes using the survival analysis methods. The cumulative incidence of long-term outcomes was assessed using the Kaplan–Meier statistics. Furthermore, the hazard ratios of long-term outcomes were calculated using the Cox regression analysis.

## Discussion

To the best of our knowledge, this study is the first research to prospectively investigate the long-term recurrence and the clinical impact of POAF using an event recorder in patients with malignancy.

Early documentation of POAF could be essential to prevent potential embolic events. In addition, underdiagnoses are often possible because AF lasts for a very short period, and an ECG monitor is the only means to provide a definitive diagnosis. To date, several risk scores and equations for the prediction of AF in the general population have been developed and validated. In this study, we will validate the efficacy of such risk scores in the setting of the perioperative period by assessing our dataset.

Even if patients with malignancy survive by overcoming the disease, they might die from any preventable cardiovascular diseases. In particular, those with POAF might develop cardiogenic stroke in the future. Because details of the natural history of patients with POAF remain unclear, investigating the need to continue anticoagulation therapy for such patients is necessary.

The apparent correlation between POAF and stroke can be explained by other atherosclerotic factors, such as age, hypertension, and diabetes mellitus [6, 7, 21]. Therefore, whether AF causes subsequent stroke remains unclear. Physicians in this study follow up patients in real time and record the incidence of POAF and any complications, which might be helpful for assuming the causal relationship between AF and morbidities, chronologically. Furthermore, the instrumental variable method, if appropriate, could reinforce the supposed correlation.

Although POAF post-non-cardiac surgery is an increasingly common problem and attracts clinicians’ attention, both short- and long-term characteristics and management have remained unclear because of limited evidence [22].

This study has some limitations. First, the present cohort incudes limited subsets of non-cardiac surgery. The results acquired from the present study may not be adapted for the other subsets, such as non-malignancy. Second, the incidence of AF in the patietns without POAF may be underestimated because they do not wear the event recorder. However, the most important question is whether we should prescribe anticoagulation for patients with POAF in future. We believe that the results of this cohort will provide several indications for the management of patients with malignancy who developed POAF.

## Additional files

Additional file 1: **Table S1.** The settings of automatic detection function (DOCX 13 kb)

Additional file 2: “Definition of outcome” and “Details of SPIDER FLASH-t AFib^®^”. (DOCX 19 kb)

## Abbreviations

AF: Atrial fibrillation
ECG: Electrocardiogram
POAF: Perioperative atrial fibrillation
