# Supplementary material for: The study protocol for PREDICT AF RECURRENCE: a PRospEctive cohort stuDy of surveIllanCe for perioperaTive Atrial Fibrillation RECURRENCE in major non-cardiac surgery for malignancy
Source: BMC Cardiovasc Disord. 2018 Jun 26;18:127. doi: 10.1186/s12872-018-0862-9 (PMC6019832; doi:10.1186/s12872-018-0862-9)
Supplement: Supplementary file 1 — Table S1. The settings of automatic detection function (DOCX 13 kb) [file 12872_2018_862_MOESM1_ESM.docx]

Table S1. The settings of automatic detection function

|  | prematurity (%) | threshold (msec) | Minimum duration (sec) | Time before (sec) | Time after (sec) |
| --- | --- | --- | --- | --- | --- |
| Supraventricular tachycardia | 75 | 375 | 15 | 60 | 60 |
| Ventricular tachycardia | 80 | 500 | 8 | 60 | 60 |
| Irregular RR interval |  |  | 30 | 60 | 120 |
| Bradycardia |  | 1500 | 20 | 60 | 60 |
| Pause |  | 2500 |  | 30 | 30 |
| Missed beats |  | 1500 |  | 30 | 30 |
