# Supplementary material for: The study protocol for PREDICT AF RECURRENCE: a PRospEctive cohort stuDy of surveIllanCe for perioperaTive Atrial Fibrillation RECURRENCE in major non-cardiac surgery for malignancy
Source: BMC Cardiovasc Disord. 2018 Jun 26;18:127. doi: 10.1186/s12872-018-0862-9 (PMC6019832; doi:10.1186/s12872-018-0862-9)
Supplement: Supplementary file 2 — “Definition of outcome” and “Details of SPIDER FLASH-t AFib®”. (DOCX 19 kb) [file 12872_2018_862_MOESM2_ESM.docx]

Prediction of recurrence of POAF

Supplementary Appendix

1. Definition of outcome

1.1. Cause of death

Causes of death are evaluated. Cardiac death is defined as death due to coronary artery disease, lethal ventricular arrhythmia, congestive heart failure, and the other heart diseases. Non-cardiac death is defined as death not caused by any heart disease. Out-of-hospital death with a presumed sudden pulseless condition and the absence of evidence of a non-cardiac condition is regarded as cardiac death. The cause of death is decided by at least two main doctors at the corresponding department.

1.2. An etiology of stroke

An etiology of stroke includes lacunar, atherothrombotic, and embolic cerebral infarction. The differential diagnosis is decided by at least two neurologists and radiologists.

1.3. Bleeding

Definition of major/minor bleeding is decided in accordance with the ROCKET AF study.[^1^](#_ENREF_1) Major bleeding is defined as clinically overt bleeding associated with any of the following: fatal outcome, involving a critical site (ie, intracranial, intraspinal, intraocular, pericardial, intraarticular, intramuscular with compartment syndrome, or retroperitoneal), or clinically overt bleeding associated with a fall in hemoglobin concentration of ≥2 g/dL, or leading to transfusion of ≥2 units of packed red blood cells or whole blood. Clinically relevant, minor bleeding is defined as overt bleeding not meeting the criteria for major bleeding but associated with medical intervention.

2. Details of SPIDER FLASH-t AFib^®^

The automatic detection of arrhythmias allows SPIDER FLASH-t AFib^®^ to record 60 seconds before and after an event in two channels. The settings of automatic detection function are shown in Supplemental Table 1. The event recorder can distinguish between P wave and QRS complex referring to QRS width. If a patient develops supraventricular tachycardia (SVT) at cycle length ≤375 milliseconds for ≥15 seconds, the 60 seconds before and after the event is recorded. If RR interval irregularity longs for ≥30 seconds, the 60 seconds before and the 120 seconds after irregularity is recorded as AF. If a patient develops ventricular tachycardia at ≤500 milliseconds for ≥8 seconds, the 60 seconds before and after the event is recorded. Premature atrial contractions within the first 75% and premature ventricular contractions within the 80% of the RR interval is recorded. Heart rate at cycle length ≥1500 milliseconds lasting for ≥20 seconds is recorded as bradycardia. Any pause at cycle length ≥2500 milliseconds is recorded the 30 seconds before and after the event. RR interval at cycle length ≥1500 milliseconds lasting for <20 seconds is recorded as missed beats the 10 seconds before and after them.

Reference

**1.** Rivaroxaban-once daily, oral, direct factor Xa inhibition compared with vitamin K antagonism for prevention of stroke and Embolism Trial in Atrial Fibrillation: rationale and design of the ROCKET AF study. *Am Heart J.* 2010;159:340-347 e341.

Supplementary Table 1. The settings of automatic detection function

|  | prematurity (%) | threshold (msec) | Minimum duration (sec) | Time before (sec) | Time after (sec) |
| --- | --- | --- | --- | --- | --- |
| Supraventricular tachycardia | 75 | 375 | 15 | 60 | 60 |
| Ventricular tachycardia | 80 | 500 | 8 | 60 | 60 |
| Irregular RR interval |  |  | 30 | 60 | 120 |
| Bradycardia |  | 1500 | 20 | 60 | 60 |
| Pause |  | 2500 |  | 30 | 30 |
| Missed beats |  | 1500 |  | 30 | 30 |
